# Effects of electroacupuncture on bladder dysfunction and the expression of PACAP38 in a diabetic rat model

**DOI:** 10.3389/fphys.2022.1008269

**Published:** 2023-01-09

**Authors:** Xuke Han, Yiding Chen, Lue Ha, Jiao Yang, Fangzhou Wang, Huizhen Chen, Qian Zhou, Cong Long, Xianliang Qiu, Qiu Chen

**Affiliations:** ^1^ College of Acupuncture and Massage, Shaanxi University of Chinese Medicine, Xianyang, China; ^2^ Department of Endocrinology, Hospital of Chengdu University of Traditional Chinese Medicine, Chengdu, China; ^3^ West China Second Hospital, Sichuan University, Chengdu, China

**Keywords:** electroacupuncture, diabetic bladder dysfunction, type 2 diabetes mellitus rats, cystometry, pituitary adenylate cyclase activating polypeptide

## Abstract

**Objective:** To explore the effects and the possible mechanism of electroacupuncture (EA) on diabetic bladder dysfunction (DBD) in streptozotocin-high fat diet (STZ-HFD) induced type 2 diabetes mellitus (T2DM) rats.

**Methods:** The experiment was divided into Control, diabetic bladder dysfunction, electroacupuncture, and Sham electroacupuncture group. After 8 weeks of electroacupuncture intervention, the body mass, 24 h urine volume, intraperitoneal glucose tolerance test (IPGTT), and urodynamics were detected. After the wet weight of the bladder was detected, the hematoxylin-eosin (HE), Masson’s trichrome, and TUNEL were used to analyze histological changes. The PACAP38 expressions in the bladder were detected by Real-time PCR and Western blot.

**Results:** Compared to the Control group, the bladder wet weight, 24 h urine volume, blood glucose, maximum bladder capacity, bladder compliance, bladder wall thickness, the smooth muscle/collagen ratio, and apoptosis rate of the diabetic bladder dysfunction group were significantly increased. Moreover, the body mass and leak point pressure were significantly reduced. Compared with the Sham electroacupuncture group, the bladder wet weight, maximum bladder capacity, bladder compliance, bladder wall thickness, and apoptosis rate of the electroacupuncture group were significantly reduced. In contrast, the leak point pressure was increased. The PACAP38 mRNA and PACAP38 protein expression of the diabetic bladder dysfunction group were significantly lower than the Control group, while electroacupuncture treatment could upregulate PACAP38 mRNA levels and PACAP38 protein expression of diabetic bladder dysfunction model rats.

**Conclusion:** electroacupuncture could ameliorate bladder dysfunction in the diabetic bladder dysfunction model rats by reversing bladder remodeling, which might be mainly mediated by regulating the PACAP38 level.

## 1 Introduction

Epidemiological studies show that about 500 million people with diabetes mellitus (DM) globally, accounting for 11% of the world’s adults; by 2040, the number of people with diabetes in the world will reach 642 million (Ramirez et al., 2021). Urinary complications are among the most common complications in diabetic patients (Boga et al., 2020). About 40%–80% of DM patients have bladder dysfunction symptoms (Han et al., 2020). As the most troublesome lower urinary tract symptom (LUTS) of DM, DBD has a substantially greater incidence than other known DM consequences, such as neuropathy and nephropathy (Daneshgari and Moore, 2006). The main clinical manifestation of DBD is abnormal bladder storage and voiding function, which is usually described as a triad of decreased sensation, increased bladder compliance and capacity, and impaired detrusor contractility (Daneshgari et al., 2009). Currently, the main clinical symptoms of DBD include voiding problems (urinary retention, frequent and urgent micturition) and storage problems (overactive bladder and urge incontinence) (Wittig et al., 2019). The DBD management strategies mainly reduce residual urine volume, alleviate urinary system symptoms, and protect renal function based on lifestyle intervention and glycemic control (Yuan et al., 2015). However, the existing behavioral and pharmacological treatments have limited effect in relieving the detrusor dysfunction of DBD patients.

Pituitary adenylate cyclase-activating polypeptide (PACAP) is a neuropeptide discovered in the hypothalamus in 1989 and belongs to the secretin/glucagon/vasoactive intestinal polypeptide family (Arimura, 1998). As a cytoprotective peptide with diverse biological functions, PACAP is widely distributed in the body, not only in the central and peripheral nervous systems but also in the pancreas, bladder, muscles, and cornea (Toth et al., 2020). Numerous studies indicate that PACAP could be used as a therapy or preventative measure for diabetes problems (Banki et al., 2013; Szabadfi et al., 2014; Marzagalli et al., 2015). According to relevant research, PACAP regulates signal molecular networks involved in neuronal protection and repair (Rivnyak et al., 2018). The PACAP/receptor system has tissue-specific distribution in the lower urinary tract (Ojala et al., 2019). As a sensory neurotransmitter in the urinary tract, PACAP mainly exists in the urinary tract in the form of PACAP38, and high-density PACAP38-positive nerve fiber bundles are widely expressed in the bladder adventitia, smooth muscle and subepithelial layers (Hernandez et al., 2006; Girard et al., 2017). Therefore, it can be speculated that the PACAP/receptor system in micturition pathways may be a potential target for bladder dysfunction treatment.

In the clinical practice of many countries, acupuncture has been widely used to treat bladder dysfunction and improve neurological function (Chan et al., 2018). Studies have reported that acupuncture can effectively inhibit overactive bladder in rats, improve bladder compliance, and reduce pathological damage to bladder tissue, thereby improving bladder function (Feng et al., 2013; Zhang et al., 2019). It has also been theorized that acupuncture may be a complementary and alternative therapy for DBD radical treatment (Tong et al., 2009). Electroacupuncture (EA) is an important method to prevent and treat DM and its complications (Yao et al., 2021). BL23, BL33, and SP6 are acupoints widely studied in DM treatment (its common chronic complication) and bladder dysfunction (Liu et al., 2017; Feng et al., 2018). BL28 corresponds to sacral vertebrae S2 and S3 levels; EA stimulation of the sacral vertebrae can inhibit an overactive bladder (Feng et al., 2013). However, the specific contributing factors and mechanisms of EA for DBD are still unknown. In this study, we chose these four acupoints for EA intervention to investigate the EA effect on urodynamics, bladder histomorphology, and PACAP38 in STZ-HFD-induced type 2 diabetes rat models.

## 2 Materials and methods

### 2.1 Animals and study design

**FIGURE 1:**
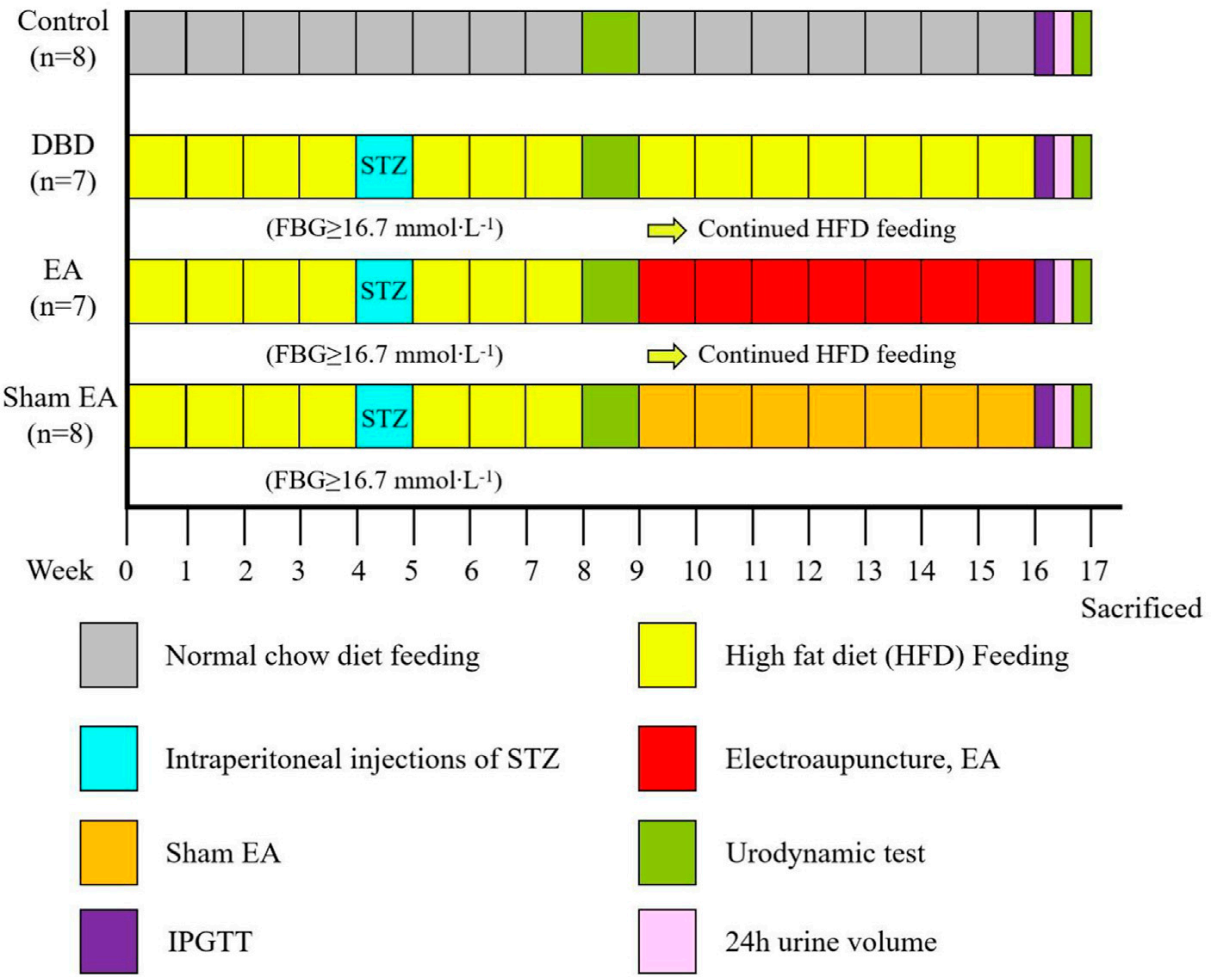
Time flow chart of rat modeling and experimental intervention. Timeline for modeling and intervention procedures.

All experiments were performed on female Sprague-Dawley rats, obtained from Chengdu Dossy Experimental Animals Company, weighing 180–220 g at the beginning of the experiment. The rats were housed in a 12 h light/dark cycle with a room temperature of about (22 ± 2)°C, relative humidity of 50%–60%, food and water available *ad libitum*. Four animals were housed in each cage. All experimental animal protocols were based on international guidelines and approved by the Ethics Committee of Chengdu Dossy Experimental Animals Company. The research time flow chart is shown in Figure 1.

First, all the rats (*n* = 48) were randomly divided into two groups: Control (*n* = 8) and Model (n = 40). The model rats were fed with HFD for 4 weeks to induce insulin resistance. After 24 h of fasting, the diabetic model rats were induced by a single intraperitoneal injection of STZ (35 mg/kg) diluted in 0.1 mol/L citrate buffer solution. After 72 h, the fasting blood glucose (FBG) of the rats’ tail vein was measured, and the serum glucose level was ≥16.7 mmol × L^−1^ for three consecutive days, indicating that the T2DM model rats were successfully established (Xu et al., 2022). The HFD feeding was continued until week eight, and models were assessed by a urodynamic test. The DBD model was established when rats exhibited irregular micturition patterns and significantly increased bladder capacity (Leiria et al., 2011). The success models (*n* = 22) were randomly divided into three groups: DBD (*n* = 7), EA (*n* = 7) and Sham EA group (*n* = 8).

After 8 weeks of intervention, the body mass, 24 h urine output, and IPGTT were detected. After the urodynamic test, the rats were sacrificed, and the bladder wet weight was detected. The bladder wall thickness was observed by HE staining, the fiber deposition was observed by Masson’s trichrome staining, and the detrusor cells’ apoptosis was analyzed by TUNEL staining. The expressions of PACAP38 in bladder were detected by real-time PCR and Western blot.

### 2.2 Acupuncture treatment

In the EA group, the disposable sterile stainless steel acupuncture needles (0.25 × 25 mm) were inserted bilaterally at BL23, BL28, and BL33 to a depth of approximately 5 mm and vertically inserted at SP6 to a depth of approximately 3 mm bilaterally. The acupuncture needle is connected to the EA stimulation apparatus (SDZ-V; Hua Tuo Medical Technology Co.). The stimulation parameters are sparse and dense waves (sparse wave 10 Hz, dense wave 50 Hz). The intensity was adjusted from 0.1 mA, using the slight trembling of rats’ limbs, squeak absence, and tolerable threshold as the criterion. Intervention for 30 min, five times a week, for eight consecutive weeks. In the Sham EA group, the acupuncture needles were superficially punctured and fixed at the same position on the body surface of the EA group. However, the acupuncture needles did not penetrate the acupoints, and the electrostimulation apparatus was turned off. The needle retention time and intervention duration in the Sham EA group were the same as in the EA group. The acupoints for EA in this study are BL28 and BL33, commonly used in clinical practice at the level of the sacral vertebra.

### 2.3 24 h urine volume measurement

After the intervention, the rats were placed in the metabolic cage. One rat per cage. Rat urine was collected in a clear plastic cup. After 24 h, the rats’ urine volumes in the plastic cups were measured.

### 2.4 IPGTT

After the 24 h urine volume measurement, rats fasted for 12 h. The intraperitoneal glucose (2 mg/kg) tolerance test (IPGTT) test was performed. The blood glucose levels in the tail vein of the rats were measured by a glucometer (FreeStyle Optium Xceed, Abbott Co.) at the following time points: 0 (before glucose injection), 15, 30, 60, and 120 min after glucose injection.

### 2.5 Urodynamic test

The urodynamic test was performed at the 8 and 16 weeks, including the following parameters: basal Pressure (BP), leakage point pressure (LPP), maximum bladder capacity (MBC), and bladder compliance (BC). The rats were anesthetized by intraperitoneal injection of sodium pentobarbital (40 mg/kg), fixed in the supine position, and the abdomen was pressed to empty the bladder. After routine disinfection, the urinary catheter was slowly inserted into the rat’s bladder through the urethra. Connect the other end of the catheter to the pressure transducer and the micro-injection pump through a three-way stopcock. After the pressure curve was stable, bladder pressure was recorded. Normal saline (25°C–35°C) was injected into the bladder at the rate of 0.1 mL/min. When liquid overflow was observed at the external orifice of the urethra, the perfusion was stopped immediately. The measurement was repeated thrice for each rat, and the average value was taken.

1) BP: When the pressure waveform stabilizes, the intravesical pressure displayed before fluid perfusion is the BBP.
2) LPP: The pressure in the bladder when liquid dripping from the urethral orifice for the first time is the LPP.
3) MBC: The capacity of the bladder when the urethral orifice drips for the first time (perfusion rate × perfusion time).

### 2.6 Histological test

After the urodynamic test, the rats were euthanized, and the whole bladder was removed and weighed using an electronic balance. A portion of the bladder was fixed in 10% paraformaldehyde solution, embedded in paraffin, and sectioned. The bladder tissue sections were stained with HE or Masson’s trichrome. The ratio of smooth muscle to collagen was measured by using Masson’s trichrome staining. The bladder wall thickness was determined by HE staining. The apoptosis was determined by the terminal deoxynucleotidyl transferase-mediated dUTP biotin nick end labeling (TUNEL) method.

### 2.7 Real-time PCR

Total RNA was extracted from the bladder using TRIzol reagent (Bomei Biotechnology Co., Ltd, China). Then, cDNA syntheses were obtained from the total RNA by using PrimeScript RT reagent Kit (RR047A, Takara Biomedical Technology Co., Ltd, China). And qPCR was performed using a QuantStudio TM3 Real-Time PCR system (Thermo Scientific, United States) under the following conditions: 95°C for 30 s, 95°C for 5 s, and 56°C for 30 s, followed by a final extension step at 72°C for 30 s. The comparative Ct (2-^△△CT^) method was used to detect the relative expression levels of the target genes. Results are expressed as mRNA levels of each gene studied, and each result was normalized according to β-actin expression. According to the whole genome sequence of PACAP38 in the NCBI database, Primer Premier software was used to design and synthesize specific primers, conduct real-time PCR experiments, and optimize reaction conditions and systems. Primer pairs are listed in Table 1. The melting curves showed that the primers amplified only one specific PCR product (Supplementary Figure S1). According to MIQE guidelines, amplification efficiencies (E values) was determined by standard curve (Bustin et al., 2009). qPCR efficiency values must be within a range from 90% to 110% and with a standard curve correlation coefficient (*R* ^2^) ≥ 0.98 (Taylor et al., 2010; Bars-Cortina et al., 2019). The qPCR primer efficiency in this study fulfils the requirements (Supplementary Figures S2—S3).

**TABLE 1:** Primers sequence of PACAP38 and β-actin.

Gene	Primer (5′–3′)
PACAP38-F	agcggagcaaggttggc
PACAP38-R	gta​aag​ggc​gta​ggc​gtc​a
β-actin-F	gaa​gat​caa​gat​cat​tgc​tcc
β-actin-R	tac​tcc​tgc​ttg​ctg​atc​ca

### 2.8 Western blot

The bladder tissue proteins were prepared using the Total Protein Extraction Kit (Beyotime Biotechnology, Jiangsu, China). The protein concentration was determined using a Pierce^®^ BCA Protein Assay Kit (Thermo Fisher Scientific, Waltham, Massachusetts, United States). Membrane proteins (20 µg) were fractionated by electrophoresis through a 10% sodium dodecyl sulfate-polyacrylamide gel electrophoresis and then transferred onto polyvinylidene difluoride membranes (Sigma-Aldrich, Missouri, United States). Membranes were blocked with TBST (1x Tris-buffered saline, 0.1% Tween 20 with 5% nonfat dry milk) for 1 h and then incubated with primary antibodies overnight at 4°C. The primary antibody concentrations were PACAP38 (1: 1000, bs-0190r, Bioss) and β-actin (1: 100000, AC026, ABclonal). After overnight incubation, the membranes were washed thrice with TBST for 5 min each and then incubated with horseradish peroxidase-conjugated secondary antibodies for 3 h at room temperature, followed by three more washes with TBST for 10 min each. Finally, protein bands were detected with the enhanced chemiluminescence kit (Affinity Biosciences, Ohio, United States). Chemiluminescent signals were detected and analyzed by the ChemiDoc XRS imaging system (Bio-Rad, Hercules, California, United States).

### 2.9 Statistical analysis

The GraphPad Prism eight software was used for data processing, and all data were expressed as mean ± standard error of the mean (SEM). Student’s t-test (two tailed) was used for comparisons between two groups. One-way analysis of variance (ANOVA) was used for multi-group comparisons followed by Bonferroni *post hoc* analysis. Probability values less than 0.05 (*p* ≤ 0.05) were considered statistically significant.

## 3 Results

### 3.1 General findings

**FIGURE 2:**
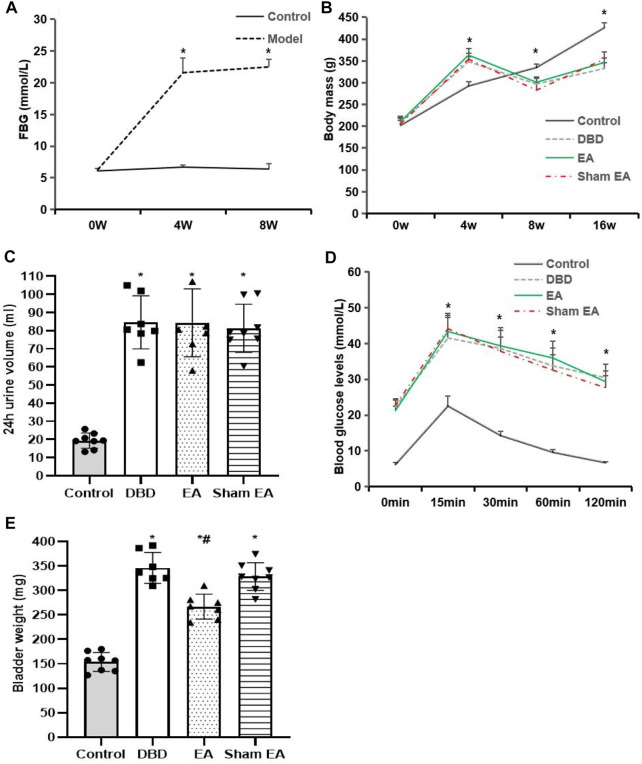
General characteristics of each group. **(A)** FBG: fasting blood glucose; **(B)** Body mass; **(C)** 24 h urine volume; **(D)** IPGTT: intraperitoneal glucose tolerance test; **(E)** Bladder weight. Control: *n* = 8 rats, DBD: *n* = 7 rats, EA: *n* = 7 rats, Sham EA: *n* = 8 rats. Results are expressed as mean ± standard error of the mean. ^*^ *p* < 0.01 *versus* the Control group, ^#^ *p* < 0.05 *versus* the Sham EA group (Student’s t-test was used to analyze the data of the FBG, and one-way ANOVA to the rest of the data).

The model rats showed a range of diabetes symptoms starting at 4 weeks. Compared with the Control group, the FBG (Figure 2A) of model rats increased gradually at 4–8 weeks, which was significantly increased at all time points compared with the Control group (4 weeks: Model 21.6 ± 2.3 vs Control 6.7 ± 0.3, 8 weeks: Model 22.5 ± 1.2 vs Control 6.4 ± 0.8, Student’s t-test, *p* < 0.01).

Compared with the Control group, the body mass (Figure 2B) of rats in the DBD group increased significantly after 4 weeks of HFD feeding (DBD 348.4 ± 10.8 vs Control 292.7 ± 10.18, One-way ANOVA, *p* < 0.01). It gradually decreased 8–16 weeks after STZ injection (8 weeks: DBD 297 ± 12.6 vs Control 335 ± 7.5, 16 weeks: DBD 332.7 ± 13.2 vs Control 426.4 ± 11.4, One-way ANOVA, *p* < 0.01). However, EA intervention did not affect the body mass of DBD rats, and there was no significant difference between the EA and Sham EA groups.

Compared with the Control group, the 24 h urine volume (Figure 2C) of all model rats in the DBD, EA, and Sham EA groups increased (all vs Control, One-way ANOVA, all *p* < 0.01), but there was no significant difference among the three groups.

In the IPGTT test (Figure 2D), the blood glucose level of all DBD model rats at each time point after glucose loading was significantly higher than in the Control group (all vs Control, One-way ANOVA, all *p* < 0.01), and the blood glucose fluctuation curve was significantly different from the Control group, indicating that the DBD model rats have impaired glucose tolerance. However, the blood glucose fluctuation curves of the rats in the EA and the Sham EA group at each time point were like the DBD group. It is speculated that EA has no obvious effect on improving the glucose tolerance of DBD rats.

Compared with the Control group, the bladder weight (Figure 2E) in the DBD, EA, and Sham EA group was significantly increased (all vs Control, One-way ANOVA, all *p* < 0.01). After 8 weeks of EA treatment, the bladder weight in the EA group was significantly lower than the Sham EA group (EA 275 ± 34.7 vs Sham EA 328 ± 46.2, One-way ANOVA, *p* < 0.05).

The results showed that the T2DM rat model was successfully induced in this experiment, indicating that the current disease status in rats is like the pathophysiological state of human T2DM combined with DBD, which is consistent with our previous findings (Han et al., 2021).

### 3.2 Effects of EA treatment on cystometric parameters

**FIGURE 3:**
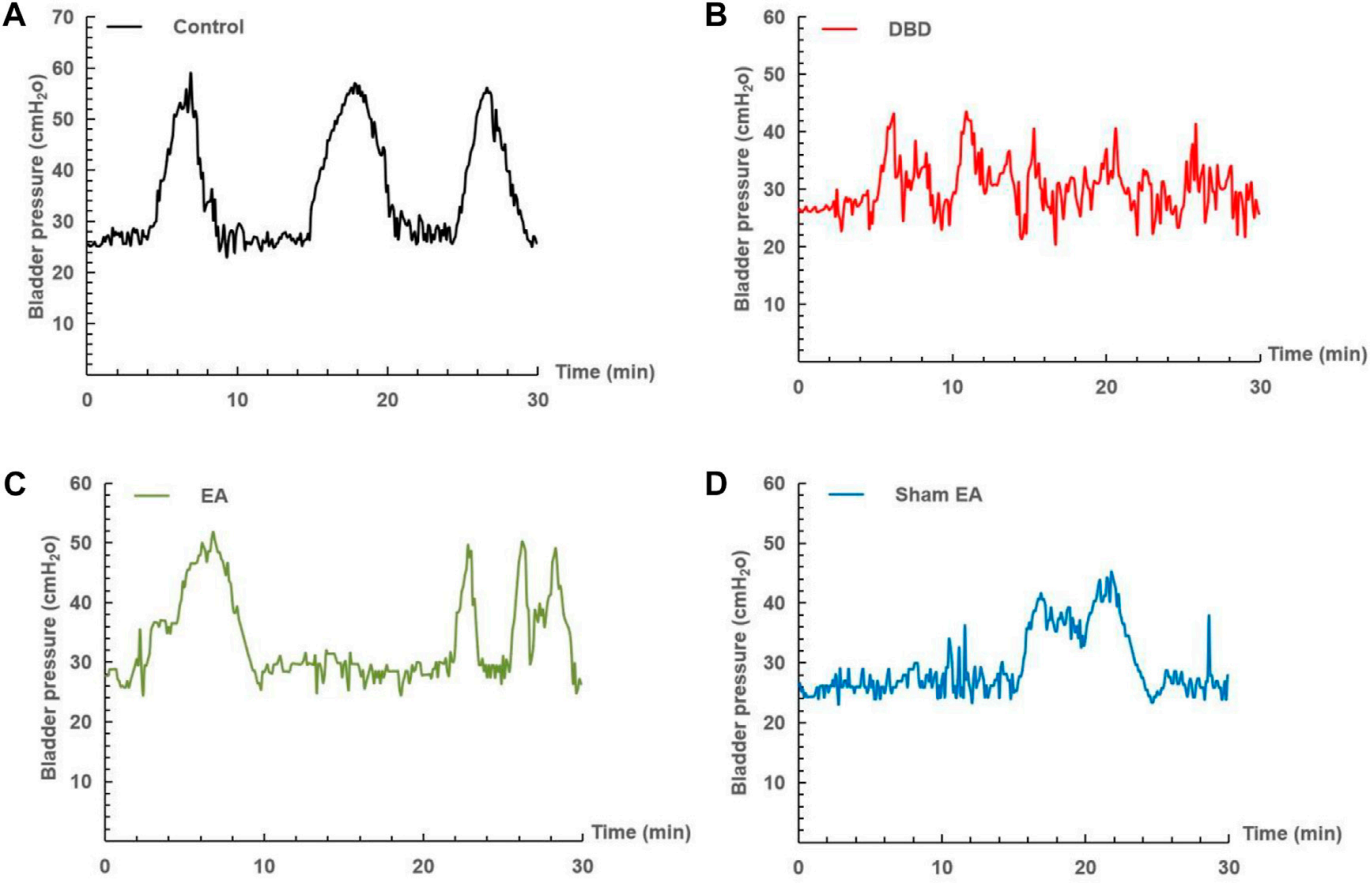
Representative cystometry records of each group. Assessment of voiding function with cystometry. EA-treated DBD model rats tended to have normal voiding patterns. **(A)** A normal voiding pattern was noted in the Control group. **(B)** An overactive bladder pattern was noted in the DBD group. **(C)** The unstable spontaneous contractile bladder pattern was noted in the EA group. **(D)** An abnormal voiding bladder pattern was noted in the Sham EA group.

The urodynamic test showed that the typical bladder pressure curve (Figure 3) differed significantly among the groups. In the Control group, the rats showed a relatively stable micturition cycle (Figure 3A). In the DBD group, the rats showed a disordered spontaneous contraction urination pattern (Figure 3B). In the EA group, the unstable pressure fluctuation of rats decreased but still showed irregular micturition intervals (Figure 3C). In the sham EA group, rats still showed disordered bladder contraction activity and abnormal micturition patterns (Figure 3D).

**FIGURE 4:**
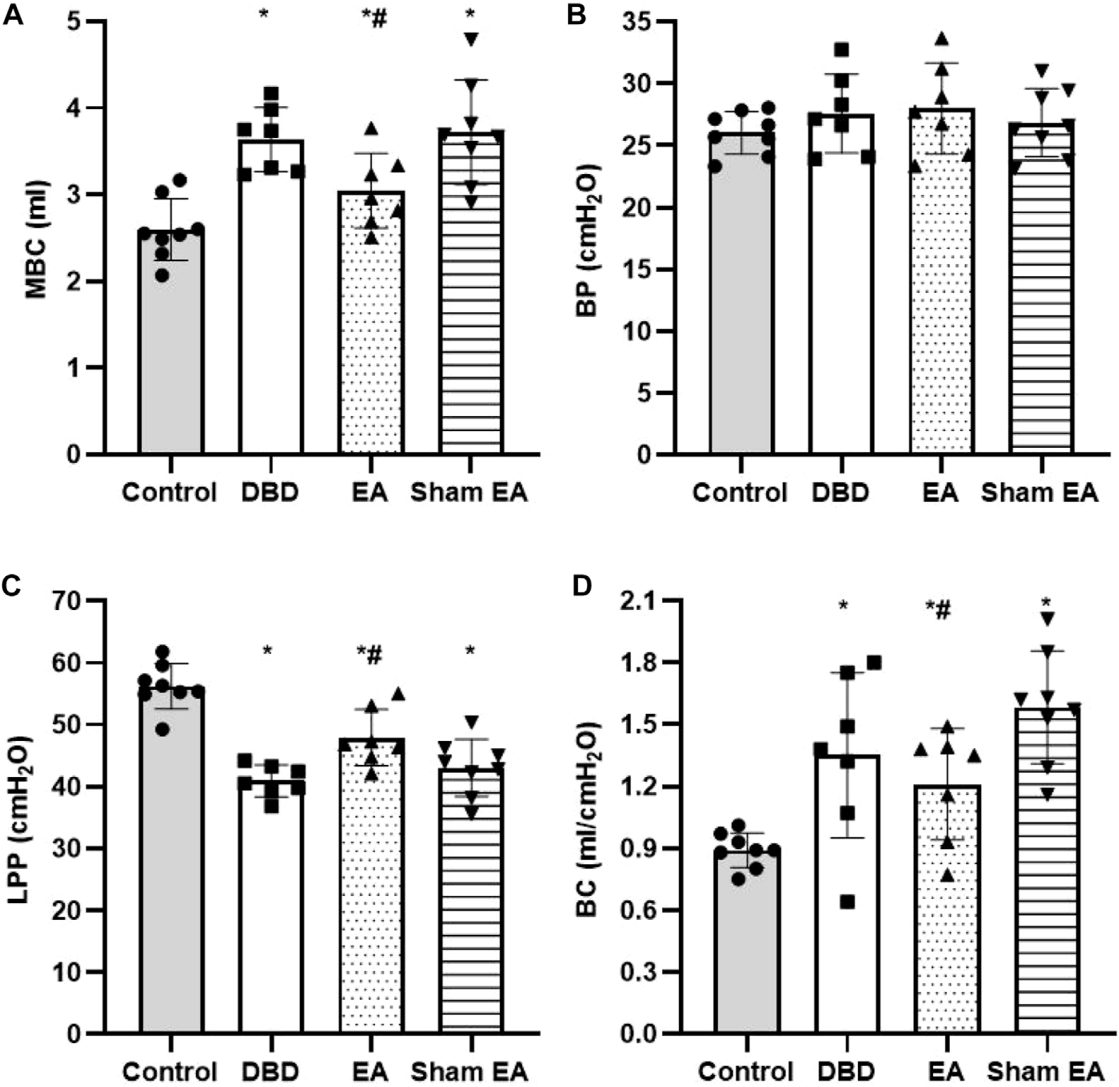
Comparison of cystometry parameters in a urodynamic test. The results of bladder wall thickness (e) in the ultrasonography test.**(A)** MBC: maximum bladder capacity; **(B)** BP: basal Pressure; **(C)** LPP: leakage point pressure; **(D)** BC: bladder compliance. Control: *n* = 8 rats, DBD: *n* = 7 rats, EA: n = 7 rats, Sham EA: n = 8 rats. Results are expressed as mean ± standard error of the mean. ^*^ *p* < 0.05 *versus* the Control group. ^#^ *p* < 0.05 *versus* the Sham EA group (one-way ANOVA).

Compared with the Control group, the MBC and BC of the rats in the DBD group were significantly increased (MBC: DBD 3.74 ± 0.43 vs Control 2.55 ± 0.48, BC: DBD 1.49 ± 0.31 vs Control 0.93 ± 0.04, One-way ANOVA, *p* < 0.05), and the LPP was decreased (DBD 40.52 ± 3.69 vs Control 55.41 ± 4.13, One-way ANOVA, *p* < 0.05). There was no statistically significant difference in BP among the groups. In this experiment, the rats’ bladder pressure is unbalanced, indicating aberrant bladder activity, and the DBD model was successfully established. Compared with the Sham EA group, the MBC and BC of the EA group decreased (MBC: EA 2.96 ± 0.27 vs Sham EA 3.67 ± 0.59, BC: EA 1.16 ± 0.23 vs Sham EA 1.57 ± 0.28, One-way ANOVA, *p* < 0.05), and the LPP increased (EA 47.92 ± 5.16 vs Sham EA 42.18 ± 3.24, One-way ANOVA, *p* < 0.05). This indicates that EA can improve the disordered micturition activity of the bladder by regulating the bladder capacity and intravesical pressure, thereby ameliorating the bladder function of the DBD model rats (Figure 4).

### 3.3 Histological findings

**FIGURE 5:**
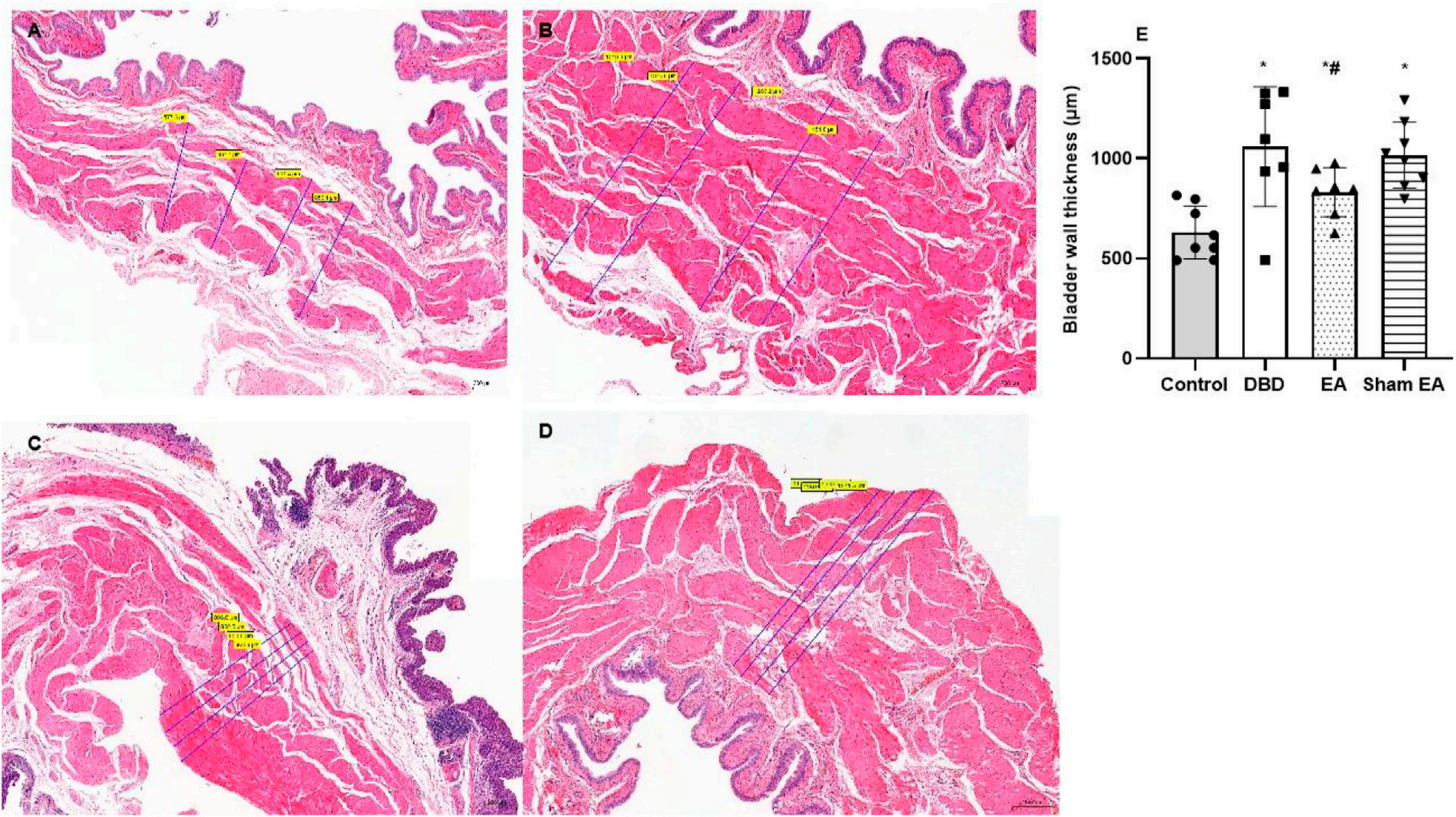
The bladder wall thickness in each group on HE images. Representative micrographs of a thin section of the bladder wall (magnification ×50). Bar graphs show the quantitative image analysis. **(A)**. Control group: n = 8 rats; **(B)**. DBD group: *n* = 7 rats; **(C)**. EA group: *n* = 7 rats; **(D)**. Sham EA group: *n* = 8 rats; **(E)**. The results of bladder wall thickness in the HE staining. Results are expressed as mean ± standard error of the mean. ^*^ *p* < 0.01 *versus* the Control group. ^#^ *p* < 0.01 *versus* the Sham EA group (one-way ANOVA).

Compared with the Control group, the bladder wall thickness (Figure 5) in the DBD group was significantly increased (DBD 1069 ± 299.9 vs Control 630.3 ± 131.7, One-way ANOVA, *p* < 0.01). This suggests that the detrusor layer of the bladder in DBD model rats is hypertrophied. Compared with the Sham EA group, the bladder wall thickness in the EA group was significantly reduced (EA 831 ± 122.4 vs Sham EA 1017 ± 165.6, One-way ANOVA, *p* < 0.01).

**FIGURE 6:**
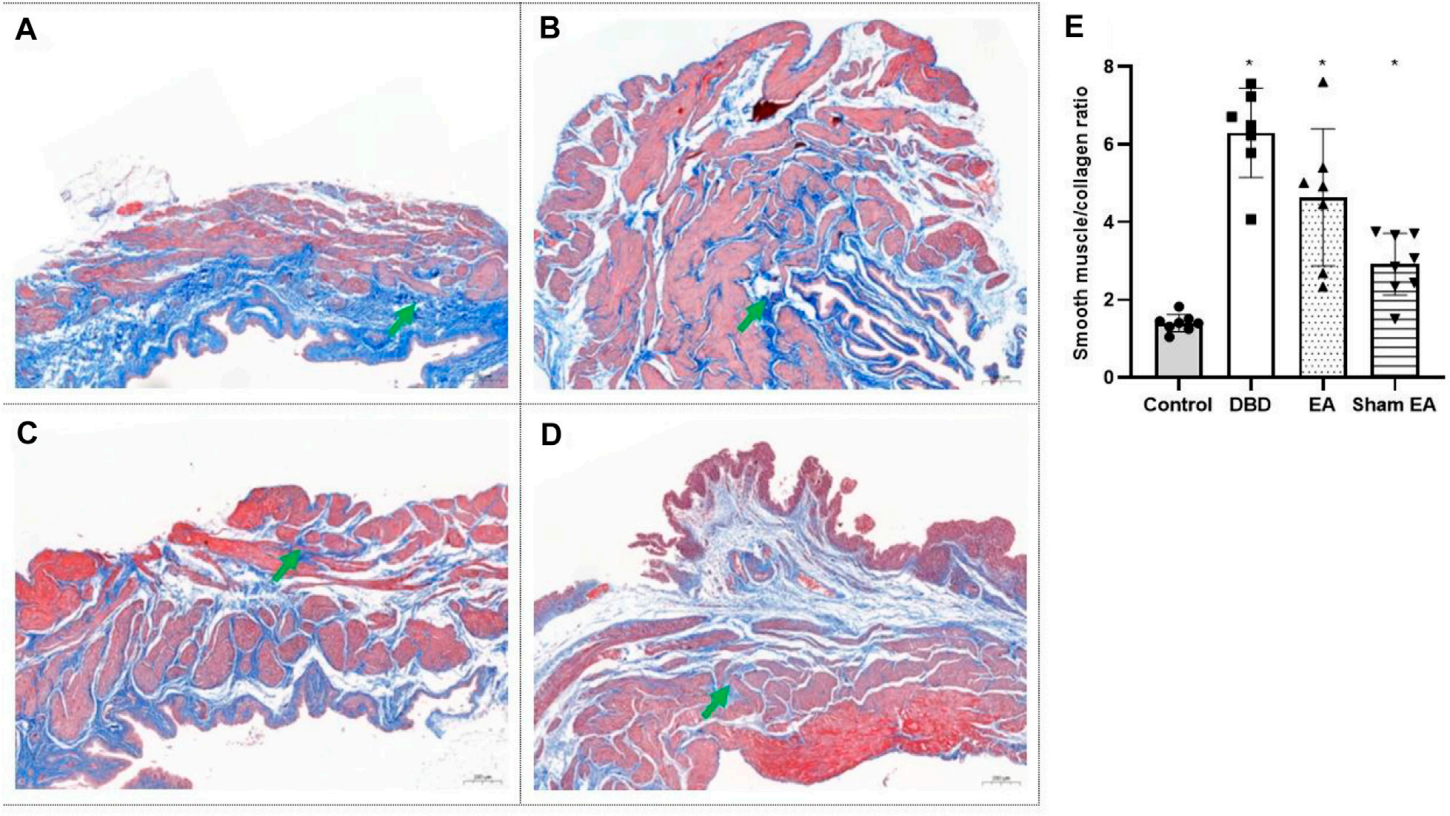
The smooth muscle/collagen ratio in each group on Masson’s trichrome images. Digitalization images (50x) from Masson’s trichrome staining. Representative micrographs show collagen with blue staining and muscle with red staining. **(A)**. Control group: n = 8 rats; **(B)**. DBD group: *n* = 7 rats; **(C)**. EA group: *n* = 7 rats; **(D)**. Sham EA group: *n* = 8 rats; **(E)**. The ratio of smooth muscle to collagen in Masson’s trichrome staining. Results are expressed as mean ± standard error of the mean. ^*^ *p* < 0.05 *versus* the Control group (one-way ANOVA). The green arrowheads indicate fiber expression.

Compared with the Control group, the ratio of smooth muscle/collagen (Figure 6) in the bladder tissues of DBD model rats (DBD, EA, and Sham EA group) was significantly increased (all vs Control, One-way ANOVA, all *p* < 0.05). However, there was no statistical difference between these three groups.

### 3.4 TUNEL findings

**FIGURE 7:**
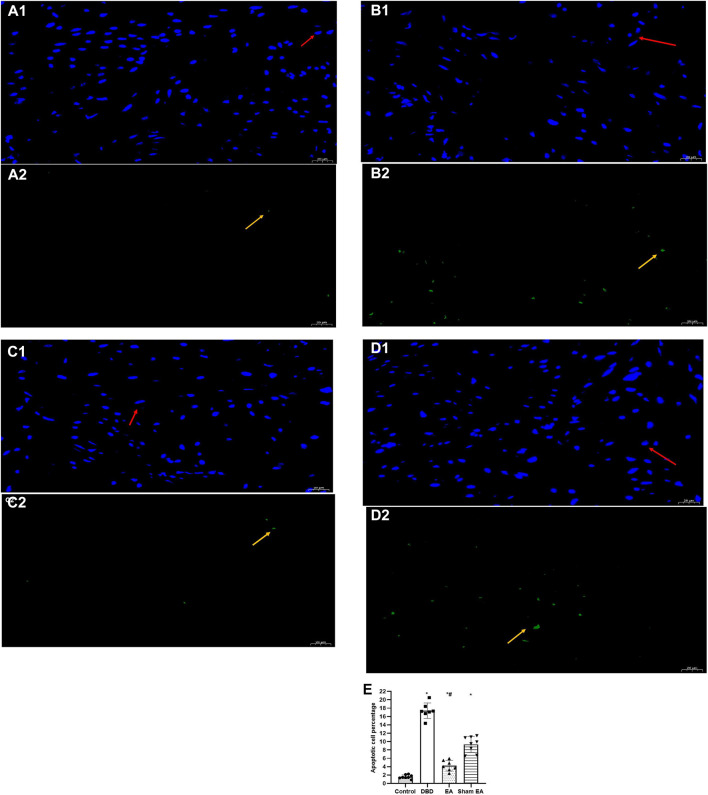
The percentage of apoptotic cells in each group on TUNEL images. Representative micrographs show TUNEL-positive cell expression: normal nuclei showed blue light, while apoptotic nuclei showed green light (magnification ×400). Bar graphs show the quantitative image analysis. **(A1-A2)**. Control group: *n* = 8 rats; **(B1-B2)**. DBD group: *n* = 7 rats; **(C1-C2)**. EA group: *n* = 7 rats; **(D1-D2)**. Sham EA group: *n* = 8 rats; **(E)**. The apoptotic cells percentage within the total number of cells in each area. Results are expressed as mean ± standard error of the mean. ^*^ *p* < 0.01 *versus* the Control group. ^#^ *p* < 0.01 *versus* the Sham EA group (one-way ANOVA). The yellow arrowheads indicate the apoptotic cells. The red arrowheads indicate normal cells.

Compared with the Control group, the apoptotic cells’ percentage (Figure 7) in the bladder tissues of the DBD group was significantly increased (DBD 17.29 ± 2.76 vs Control 1.48 ± 0.63, One-way ANOVA, *p* < 0.01). Compared with the Sham EA group, the percentage of apoptotic cells in the EA group was significantly reduced (EA 3.98 ± 1.55 vs Sham EA 9.51 ± 1.59, One-way ANOVA, *p* < 0.01).

### 3.5 Effects of EA treatment on PACAP38 level

**FIGURE 8:**
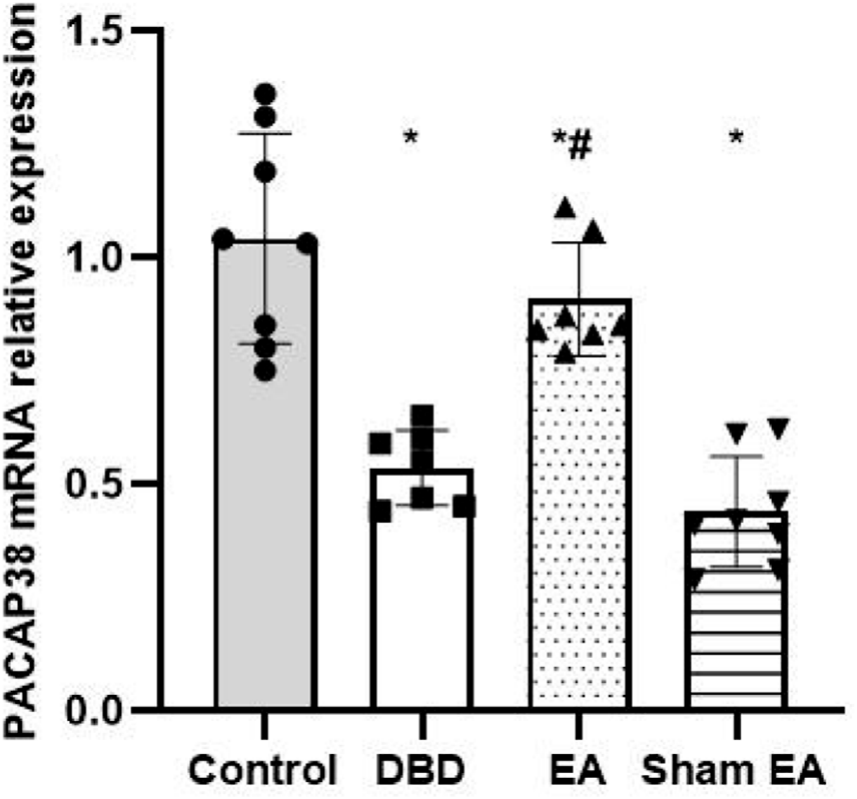
The effect of EA on PACAP38 mRNA in the rat bladder measured by real-time PCR. The relative expression of PACAP38 mRNA among groups. Control: *n* = 8 rats, DBD: *n* = 7 rats, EA: *n* = 7 rats, Sham EA: n = 8 rats. The Results are expressed as mean ± standard error of the mean. ^*^ *p* < 0.01 *versus* the Control group. ^#^ *p* < 0.01 *versus* the Sham EA group (one-way ANOVA).

The Real-time PCR experiment showed that the PACAP38 mRNA in the DBD group was significantly decreased compared with the Control group (DBD 0.55 ± 0.1 vs Control 1.03 ± 0.28, One-way ANOVA, *p* < 0.01). The PACAP38 mRNA expression was increased after EA treatment compared to the Sham EA group (EA 0.85 ± 0.26 vs Sham EA 0.46 ± 0.15, One-way ANOVA, *p* < 0.01) (Figure 8).

**FIGURE 9:**
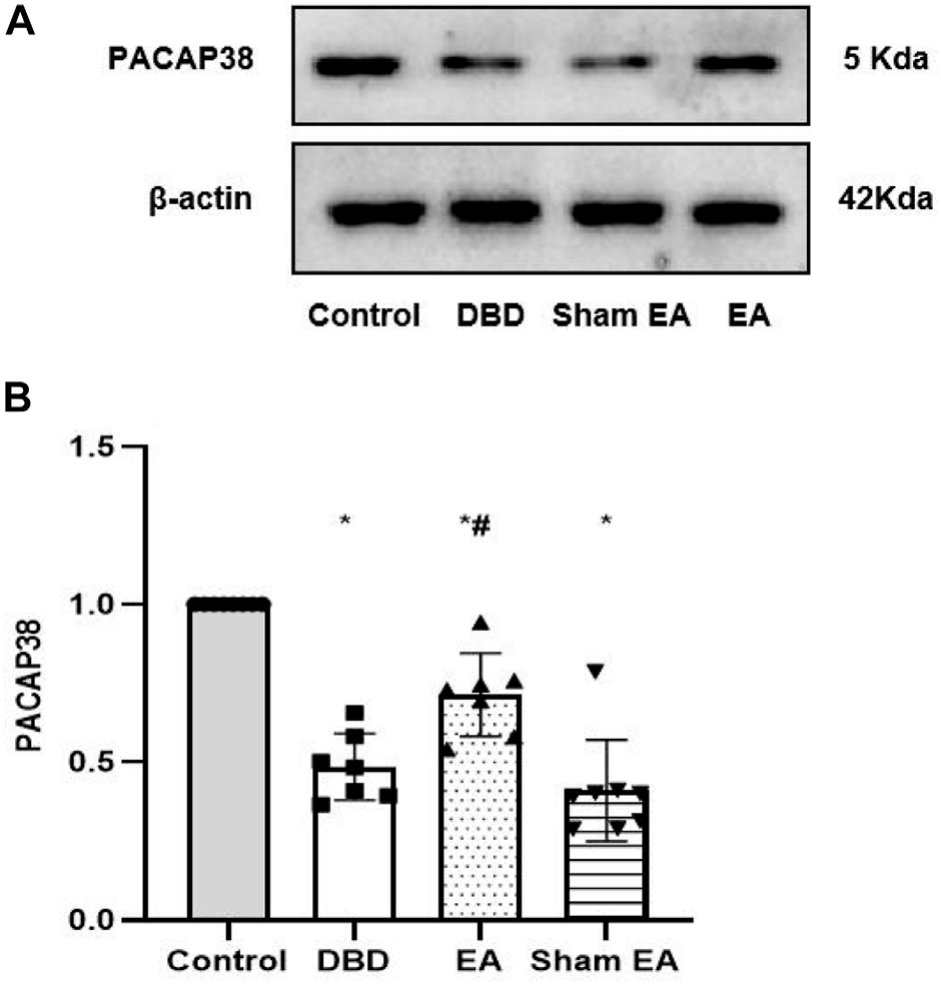
Expression of bladder PACAP38 protein in rats after the 8-week intervention **(A)**. Western blot bands of PACAP38 protein expression. **(B)**. Bar graphs show the quantitative analysis for PACAP38 protein expression. Control: *n* = 8 rats, DBD: *n* = 7 rats, EA: *n* = 7 rats, Sham EA: n = 8 rats. Results are expressed as mean ± standard error of the mean. ^*^ *p* < 0.05 *versus* the Control group. ^#^ *p* < 0.05 *versus* the Sham EA group (one-way ANOVA).

The Western blot experiment showed that the PACAP38 protein expression was significantly lower in the DBD and Sham EA groups (all vs Control, One-way ANOVA, all *p* < 0.05). Treatment with EA upregulated the PACAP38 protein expression compared to the Sham EA group (EA 0.71 ± 0.13 vs Sham EA 0.41 ± 0.16, One-way ANOVA, *p* < 0.05) (Figure 9).

## 4 Discussion

In the present study, we aimed to investigate the effects of EA treatment on bladder dysfunction and PACAP38 expression after T2DM. In previous experiments (Lee et al., 2021), low-dose STZ combined with HFD has been used to establish a T2DM model to explore the DBD development process and pathogenesis. Moreover, many promising results have been achieved. The modern definition of DBD encompasses a broad spectrum of LUTS. Due to the indistinguishable confounding factor of male benign prostatic obstruction, which has the same LUTS as DBD, it is difficult to quantify and differentiate how diabetes *per se* affects voiding dysfunction and the DBD severity. Therefore, some studies only focus on female diabetic patients after excluding other causes of lower urinary tract dysfunction (Yuan et al., 2015). Aging accelerates bladder dysfunction progression. Bladder capacity, which may increase with bladder weight and collagen deposition, will not occur quickly in SD female rats before 24 months (Chen et al., 2019). Considering the difference in physiological structure between female and male rats, the urethral resistance of male rats was greater than female rats. Female rats have a short urethra, making bladder perfusion intubation easier. Furthermore, there is no proof that gender-related differences in the structural or functional alterations may occur in the bladder of STZ-induced diabetic model rats (Rizk et al., 2006). Therefore, female SD rats were chosen for this study based on the considerations above.

DBD is characterized by raised blood glucose levels and increased bladder weight, according to a study employing STZ injection paired HFD to develop T2DM in rats (Klee et al., 2019). In this experiment, the bladder weight, 24 h urine volume, and FBG of the model rats increased, and the body mass decreased, all of which indicated that the model rats had bladder damage, detrusor dysfunction, compensatory bladder hypertrophy, and effectively reproduced DBD-related symptoms. In line with the typical clinical pathogenesis and physiological characteristics of human DBD. According to the “time theory” of the current DBD research: the changes of urodynamic indicators in DBD patients are time-dependent, with bladder compensation (overactive bladder symptoms) in the early phage and decompensation (underactive bladder activity) in the late phase (Daneshgari et al., 2009). The cystometry results of our model rats were consistent with an overactive bladder, presumably more in line with early-stage DBD.

Currently, it is acknowledged that bladder remodeling, which includes the alteration of the matrix composition of the bladder wall and the hypertrophy of the bladder detrusor, is the most important change associated with DBD (Su et al., 2004). Morphological changes in bladder remodeling include increased bladder wall thickness, contractility, bladder compliance, and apoptosis rates in detrusor smooth muscle and urothelium. Moreover, reorganizing structural relationships among the detrusor, urothelium, and collagen may be a significant factor in early-stage compensated bladder function in diabetics (Pitre et al., 2002; Liu and Daneshgari, 2006). Relevant studies have reported changes in bladder weight (bladder hypertrophy) and bladder components (smooth muscle content increased, collagen decreased) in diabetic rats (Eika et al., 1994; Xiao et al., 2015). Relevant studies found that the detrusor muscle layer in diabetic rats was significantly thickened, and the smooth muscle-to-collagen ratio of the bladder was significantly increased, consistent with our results (Masuda et al., 2020). This study found that the 24 h urine volume, bladder wall thickness, ratio of smooth muscle/collagen, and bladder cell apoptosis of DBD model rats were significantly increased, indicating that DBD model rats exhibited pathological tissue structure damage caused by the continuous influence of various harmful factors. It is speculated that EA can reduce bladder cell apoptosis, relieve bladder wall thickening, improve bladder hypertrophy caused by polyuria, and reverse bladder remodeling.

Hallmark clinical features of DBD include increased MBC and BC and decreased LPP (Ellenberg, 1980; Xiao et al., 2013; Wang et al., 2018). This is consistent with the results of this study. Compared with the Control group, the MBC and BC of the DBD group were significantly increased, and the LPP was decreased, suggesting that the DBD model rats’ bladder function had degenerated. With the DBD progression, Na^+^-K^+^-ATP enzyme metabolism was disordered, aggravating cell metabolism disorders/apoptosis, resulting in decreased bladder sensation and increased MBC. After bladder overactivity during the compensatory phase, the abnormal metabolic function of detrusor muscle cells leads to decreased contractility of the bladder detrusor, which may turn to the decompensated phase and increase BC. After EA treatment, the MBC and BC of DBD model rats decreased, and LPP increased, indicating that EA treatment improved the condition of high bladder compliance in the DBD model rats. Changes in the ratio of smooth muscle to collagen and bladder wall hypertrophy will alter BC (Levin et al., 1995; Liu and Daneshgari, 2006). A study on female diabetic rats reported that the disproportionate increased ratio of smooth muscle/collagen contributes to increased BC (Xiao et al., 2013). This study observed an increase in smooth muscle proportion resulting in a decrease in collagen percentage, likely contributing to the higher BC in DBD rats. EA could downregulate the bladder weight and wall thickness of DBD rats to balance the intravesical pressure. Moreover, BC regulates the bladder micturition reflex, loosens detrusor muscle, and improves bladder function. The bladder has two functions: urine storage and voiding (Coolen et al., 2019). Intravesical pressure is generally maintained at a relatively low level without detrusor contraction until the urine volume reaches MBC during the storage phase. In this experiment, the cytometry showed that the DBD rats frequently appeared irregular small pressure fluctuations, indicating that the DBD model rats had disordered spontaneous contractions before urination. However, the micturition threshold of rats in the EA group recovered significantly, and small pressure fluctuations decreased. Furthermore, the pressure curve tended to be stable, indicating that EA treatment reduced the detrusor muscle’s unstable spontaneous contraction activity.

Studies have found that PACAP signaling is vital in the sensory components of the bladder (bladder afferent nerves, urothelial cells) and helps regulate bladder voiding reflex function (Heppner et al., 2019). Furthermore, PACAP could regulate the urothelium solute permeability and directly control the contractility and activity of the bladder (Braas et al., 2006). Consequently, these data suggest that unbalanced PACAP38 levels coupled with bladder remodeling may be a pathophysiological mechanism in DBD. Our previous study confirmed that EA could effectively alleviate bladder dysfunction in diabetic rats (Han et al., 2021). Moreover, another study found that EA can regulate the urodynamics of neurogenic bladder model rats by up-regulating the protein expressions of PACAP38 and PAC1-R (Liu et al., 2017).

Further research showed that the bladder weight of PACAP knockout mice increased, that the bladder lamina propria and detrusor smooth muscle were significantly thickened, that the mice showed an increase in bladder capacity, voiding volume, and residual volume, and that their inter-contraction interval and detrusor contraction duration were significantly lengthened (Coolen et al., 2019). Our findings are consistent with current evidence that impairment of the PACAP expression leads to changes in bladder morphology and function and somatic and visceral sensation. The real-time PCR and western blot results revealed that EA could significantly increase PACAP38 mRNA and PACAP38 protein expression in the bladder of DBD model rats.

Hyperglycemia and peroxide toxin caused by DM destroy the function of cells and smooth muscles, resulting in the imbalance of muscle coordination of the urinary function, leading to DBD (Lee et al., 2021). This also confirms our research results. We speculate that the EA mechanism in DBD treatment is as follows: (Ramirez et al., 2021). EA can reduce the bladder cell apoptosis of DBD model rats, alleviate the pathological condition of bladder tissue, improve bladder remodeling, and repair the damaged bladder tissue morphology. (Boga et al., 2020). EA can coordinate the rhythmic relaxation and urinary function muscle contraction to improve the bladder wall thickening caused by compensatory hypertrophy, reduce the bladder weight, and improve the voiding dysfunction of DBD. (Han et al., 2020). EA can improve bladder dysfunction by regulating the PACAP38 level in the bladder of DBD model rats. Due to the complex nature of DBD, further study may be necessary to elucidate the mechanism of EA treatment. According to reports, stimulation of the PACAP/receptor system starts signaling cascades in smooth muscle, affects the cyclic adenosine monophosphate-protein kinase A (cAMP-PKA) pathway, and modifies smooth muscle contractility to relax the bladder neck (Hernandez et al., 2006; Yoshiyama and de Groat, 2008). It was found that PACAP38 can activate the PAC1 receptor of urothelial cells to release ATP to activate the purinergic receptor on the detrusor, which proves that PACAP signaling regulates bladder detrusor function by up regulating cAMP level (Girard et al., 2008). Therefore, further studies will focus on PACAP expression and cAMP-PKA signal pathways in the bladder after EA, which may explore new mechanisms of EA for DBD.

This study also had several limitations. First, we did not directly examine the contractility of bladder detrusor strips. Since the *in vitro* experiment of bladder smooth muscle contractility is not affected by factors such as neurohumoral fluids, it can intuitively reflect the changes in the contractility of the bladder detrusor strip itself and the effect of exogenous substances on it (Kullmann et al., 2014). Therefore, further research should be conducted on the intervention of exogenous substances related to the PACAP-related signal pathways to clarify the regulatory effect of EA on detrusor contraction in the DBD animal model and its specific physiological and pathological mechanism. Second, future studies need to increase the sample size and establish appropriate acupoint control groups to distinguish acupoint-specific, electrical stimulation, and non-specific acupuncture effects.

## 5 Conclusion

EA may alleviate bladder dysfunction and reverse bladder remodeling by downregulating bladder cell apoptosis and upregulating PACAP38 in DBD model rats.

## Data Availability

The raw data supporting the conclusion of this article will be made available by the authors, without undue reservation.
