# Supplementary figures and images for: Effects of electroacupuncture on bladder dysfunction and the expression of PACAP38 in a diabetic rat model
Source: Front Physiol. 2023 Jan 9;13:1008269. doi: 10.3389/fphys.2022.1008269 (PMC9868671; doi:10.3389/fphys.2022.1008269)

Figure S2. The PACAP38 real-time PCR standard curve.

E= 94.462%,  $R^2 = 0.985$ , Slope = -3.462, y-int=31.840.

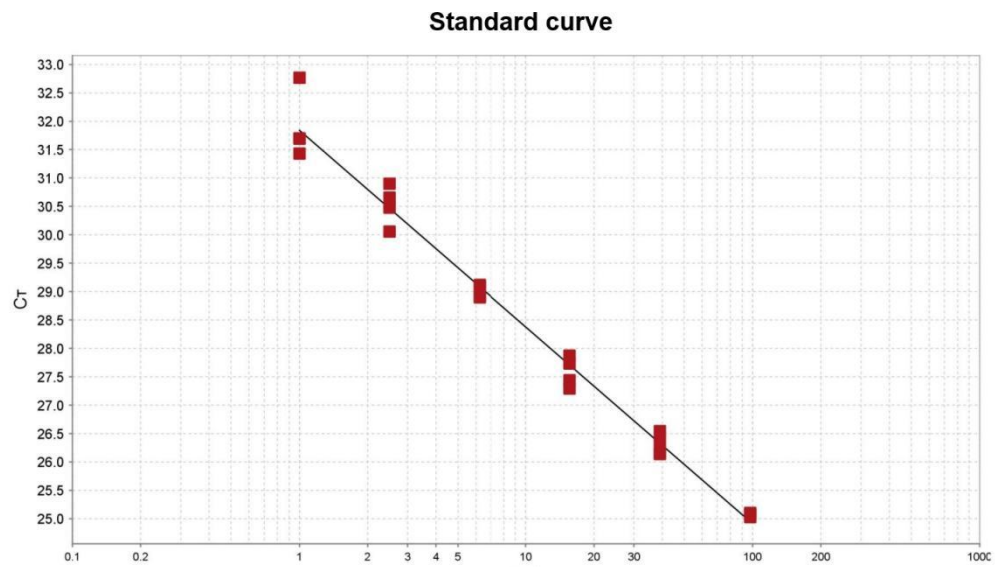

Supplement: Supplementary file 1 [file DataSheet2.PDF]

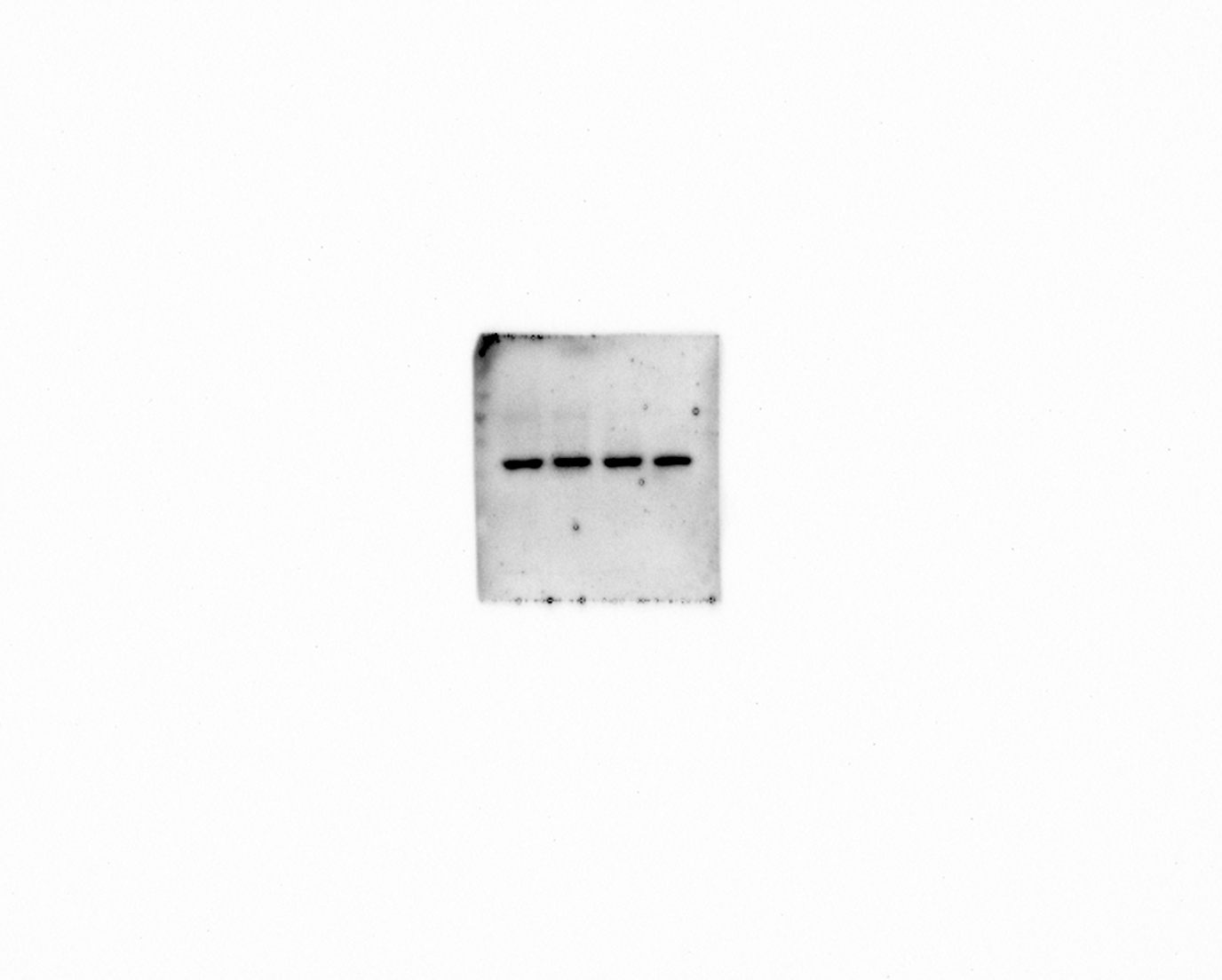

Supplement: Supplementary file 2 [file Image2.TIF]

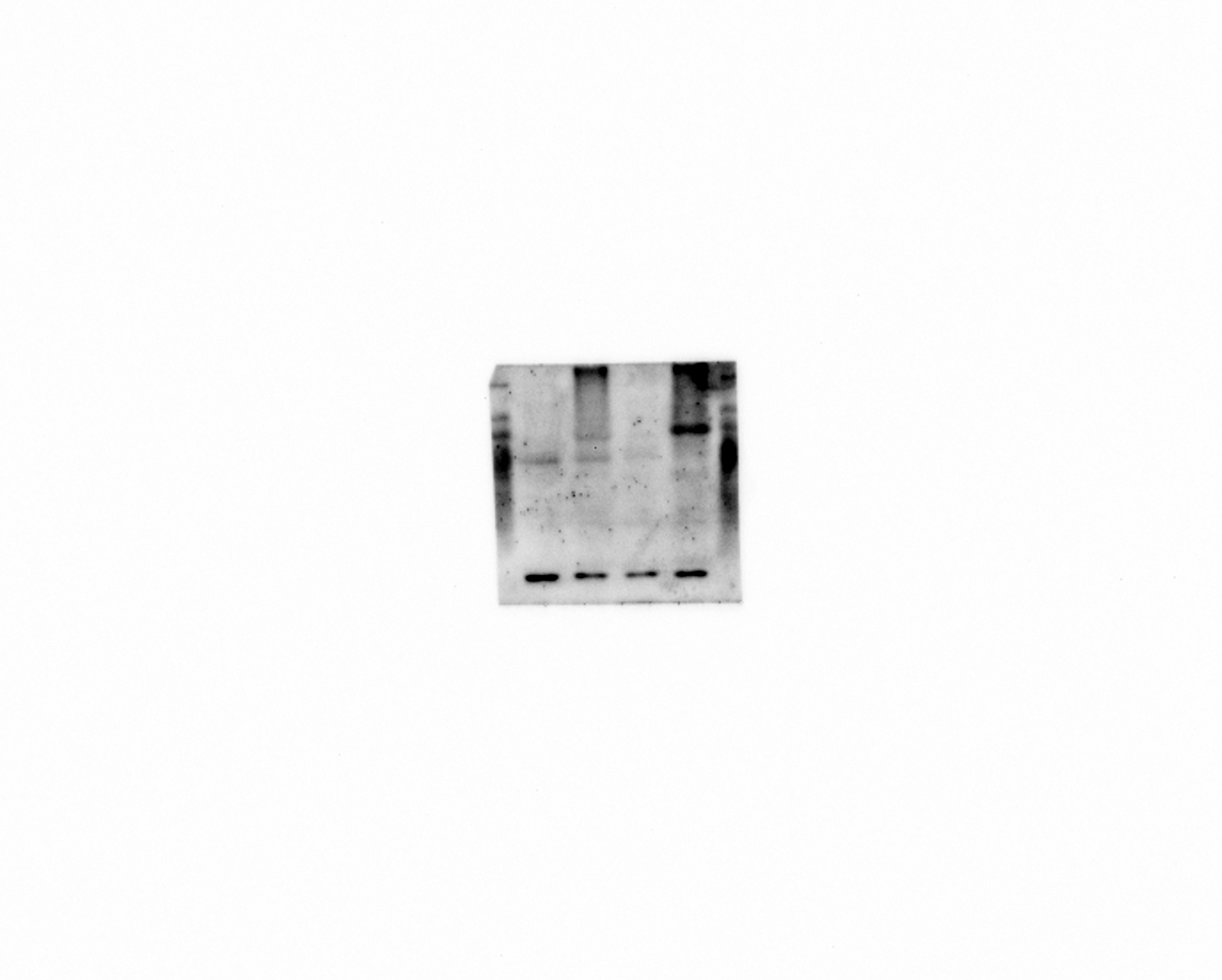

Supplement: Supplementary file 3 [file Image1.TIF]

Figure S3. The  $\beta$ -actin real-time PCR standard curve.

$E = 104.441\%$ ,  $R^2 = 0.980$ , Slope = -3.220, y-int = 24.293.

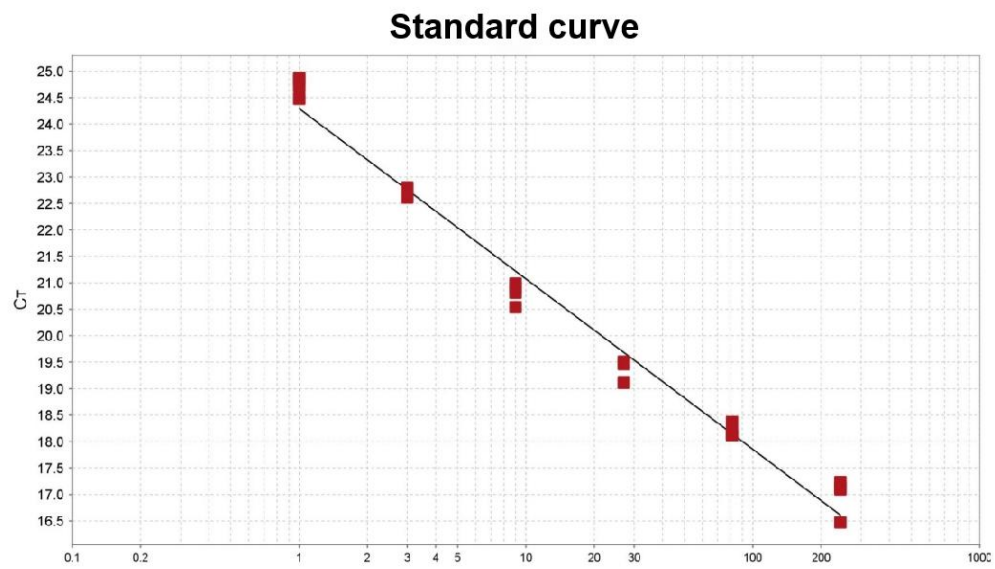

Supplement: Supplementary file 4 [file DataSheet3.PDF]
