# Supplementary material for: Effects of electroacupuncture on bladder dysfunction and the expression of PACAP38 in a diabetic rat model
Source: Front Physiol. 2023 Jan 9;13:1008269. doi: 10.3389/fphys.2022.1008269 (PMC9868671; doi:10.3389/fphys.2022.1008269)
Supplement: Supplementary file 5 [file DataSheet1.PDF]

Figure S1. The collection of PACAP38 real-time PCR amplification curves and melting curves.

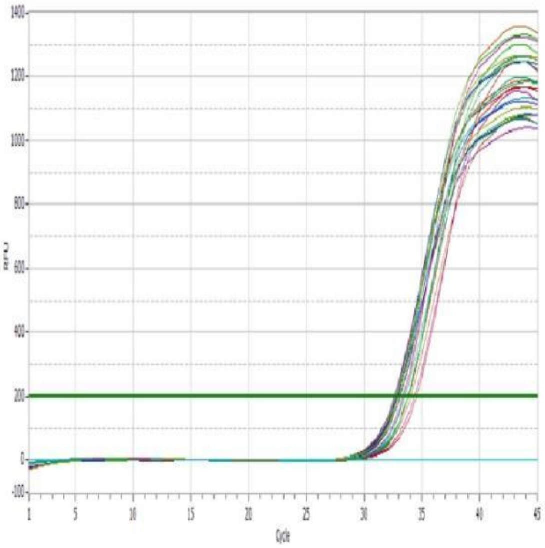

PACAP38 amplification curves

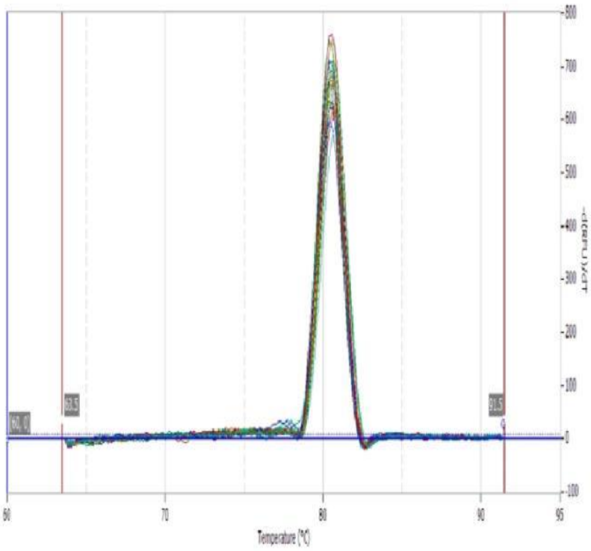

PACAP38 melting curves
